# Lysophosphatidylcholine and Carotid Intima-Media Thickness in Young Smokers: A Role for Oxidized LDL-Induced Expression of PBMC Lipoprotein-Associated Phospholipase A2?

**DOI:** 10.1371/journal.pone.0083092

**Published:** 2013-12-17

**Authors:** Anna Fratta Pasini, Chiara Stranieri, Andrea Pasini, Paola Vallerio, Chiara Mozzini, Erika Solani, Mattia Cominacini, Luciano Cominacini, Ulisse Garbin

**Affiliations:** Section of Internal Medicine D, Department of Medicine, University of Verona, Verona, Italy; University of Lille Nord de France, France

## Abstract

**Background:**

Although cigarette smoking has been associated with carotid intima-media thickness (CIMT) the mechanisms are yet not completely known. Lysophosphatidylcholine (lysoPC), a main product of lipoprotein-associated phospholipase A2 (Lp-PLA2) activity, appears to be a major determinant of the pro-atherogenic properties of oxidized LDL (oxLDL) and to induce proteoglycan synthesis, a main player in intimal thickening. In this study we assessed whether cigarette smoking-induced oxidative stress may influence plasma Lp-PLA2 and lysoPC and Lp-PLA2 expression in peripheral blood mononuclear cells (PBMC), as well as the relationship between lysoPC and CIMT.

**Methods/Results:**

45 healthy smokers and 45 age and sex-matched subjects participated in this study. Smokers, compared to non-smokers, showed increased plasma concentrations of oxLDL, Lp-PLA2 and lysoPC together with up-regulation of Lp-PLA2 (mRNA and protein) expression in PBMC (P<0.001). Plasma Lp-PLA2 positively correlated with both lysoPC (r=0.639, P<0.001) and PBMC mRNA Lp-PLA2 (r=0.484, P<0.001) in all subjects. Moreover CIMT that was higher in smokers (P<0.001), positively correlated with lysoPC (r=0.55, P<0.001). Then in *in* *vitro* study we demonstrated that both oxLDL (at concentrations similar to those found in smoker’s serum) and oxidized phospholipids contained in oxLDL, were able to up-regulate mRNA Lp-PLA2 in PBMC. This effect was likely due, at least in part, to the enrichment in oxidized phospholipids found in PBMC after exposure to oxLDL. Our results also showed that in human aortic smooth muscle cells lysoPC, at concentrations similar to those found in smokers, increased the expression of biglycan and versican, two main proteoglycans.

**Conclusions:**

In smokers a further effect of raised oxidative stress is the up-regulation of Lp-PLA2 expression in PBMC with subsequent increase of plasma Lp-PLA2 and lysoPC. Moreover the correlation between lysoPC and CIMT together with the finding that lysoPC up-regulates proteoglycan synthesis suggests that lysoPC may be a link between smoking and intimal thickening.

## Introduction

Cigarette smoking is a major risk factor for atherosclerotic vascular disease and it is an important factor contributing to premature vascular aging [1-3]. Emerging evidence demonstrates that oxidative stress and activation of inflammatory pathways contribute to many of the pathophysiologic manifestations of cigarette smoking [2-6]. Free radicals from cigarette smoking also cause peroxidation of the polyunsaturated fatty acids of cell membranes that amplify oxidative stress during smoking [5,6].

Lipoprotein-associated phospholipase A2 (Lp-PLA2) is an emerging and independent risk factor for coronary and carotid artery disease [7], that in animal studies has been shown to be involved in the causal pathway of the intimal oxidative-inflammatory cascade typical of atherogenesis [8]. In particular, a positive association between Lp-PLA2 activity and circulating oxidized low-density lipoprotein (oxLDL) has been shown in a hypercholesterolemic swine model of atherosclerosis [9]. Moreover, it has been recently reported that oxLDL can up-regulate the expression of Lp-PLA2 in THP1 monocyte-like cells and in human monocyte-derived macrophages [10]. Lp-PLA2 that is secreted predominantly by inflammatory cells, circulates in plasma mainly bound to LDL [8]. The oxidation of LDL within the arterial wall, a key factor in atherogenesis, yields oxidized phospholipids that are the substrate for Lp-PLA2 reaction, producing lysophosphatidylcholine (lysoPC) and oxidized free fatty acids both of which exhibit several pro-atherogenic effects [8,11]. *In vitro* observations have demonstrated that lysoPC is a major determinant of the pro-atherogenic properties of oxLDL and activates the redox-sensitive inflammatory pathways by increasing the recruitment of leukocytes and by inducing endothelial dysfunction and apoptosis of endothelial and vascular smooth muscle cells (SMCs) [8,11].

Ultrasound-measured carotid intima-media thickness (CIMT) represents a measure of early alterations of the arterial walls, that has been widely used as a markers of subclinical atherosclerosis and a predictor of future cardiovascular disease events [10,12,13]. In this context, however, the accuracy of CIMT as a marker of atherosclerosis is mired by the fact that main predictors of medial hypertrophy or intimal thickening are age and hypertension [14]. Moreover, in clinical cardiology, smokers often present (at a younger age) myocardial infarction without extensive visible coronary atherosclerosis [2,15]. Although epidemiological studies have consistently shown that cigarette smoking is associated with higher CIMT [16,17], the mechanisms are likely multifaceted and not yet fully understood. It has been previously suggested that cigarette smoking has a specific fibrogenic effect which causes intimal thickening [3,4]. “Secretory” vascular SMCs, the major cellular component of the neointima, secrete predominantly the small chondroitin sulfate/dermatan sulfate proteoglycans biglycan and decorin, as well as versican and the heparan sulfate proteoglycans perlecan [18,19]. The retention of LDL via their interaction with biglycan and versican at sites of intimal thickening is believed to be a key step in early atherogenesis [20-22]. Interestingly, oxLDL was demonstrated to up-regulate biglycan synthesis in vascular SMCs, resulting in enhanced lipoprotein-proteoglycans binding [23]. Moreover, among the several bioactive lipids contained in oxLDL, only lysoPC was shown to increase biglycan synthesis [24], through reactive oxygen species formation [25].

Then, since *in vitro* studies have suggested that oxLDL increase Lp-PLA2 expression and that lysoPC may have a role in intimal expansion [24], this study aimed to assess whether cigarette smoking-induced oxidative stress affects Lp-PLA2 expression in peripheral blood mononuclear cells (PBMC) and hence Lp-PLA2 and lysoPC plasma concentrations, as well as the relationship between lysoPC and CIMT in otherwise healthy smokers.

## Methods

### Ethics statement

The study was approved by the Ethical Committee of the University of Verona and all participants provided written consent prior to commencing the study.

### Study Population

Ninety (48 males and 42 females) healthy subjects, 23–48 years of age (mean 35.8±6.2 years) were enrolled in the study. None of the participants had a history of hypertension, hypercholesterolemia or diabetes mellitus and no medications were being taken. Of the 90 subjects, 45 were active cigarette smokers with a mean number of 24.5±8.0 cigarettes/day, a mean duration of smoking of 16.5±4.2 years, a mean cumulative cigarette consumption of 17.7±5.2 packs/years and were thus assigned to the smoking group. The remaining 45 subjects did not have a history of cigarette smoking and were assigned to the non-smoking group.

### CIMT measurement

CIMT is evaluated as distance between the leading edge of the lumen-intima interface and the leading edge of the media-adventitia interface [26]. The same investigator, unaware of the characteristics of the subjects, performed non-invasive measurements of CIMT using a high-resolution B-mode ultrasound (Envisor, Philips) equipped with a 7.5-to 12-MHz linear array transducer imager. For all subjects, CIMT was measured in the far wall of the common carotid artery (within 1 cm proximal to the carotid bulb), at the bulb and the origin of the internal carotid artery of both sides [26]. On each arterial site, mean CIMT values (as averaged across the entire distance) and maximal CIMT values were recorded using Carotid Studio, a validated real-time automatic technique for CIMT measurement [27]. These 6 combined mean and maximal CIMT far-wall measurements from left and right side were averaged. 

### Blood samples and PBMC isolation

Venous blood samples were obtained from each subject after 12 hours fasting. Multiple aliquots of plasma were added with antioxidant 2,6-di-tert-butyl-4-methylphenol 10 mmol/l (Sigma) to inhibit lipid peroxidation and stored at -80° C. PBMC were isolated as previously described [28]. Briefly, whole blood was layered onto a sterile aqueous medium containing ficoll and sodium diatrizoate at a predetermined density of 1.007 g/ml at 25° C. Gentle centrifugation at room temperature resulted in the separation of PBMC at the blood/ficoll interface, with the other white and red blood cells passing through the interface. Total cholesterol, high density lipoprotein (HDL) cholesterol, LDL cholesterol, triglycerides and glucose were measured with standard methods. 

### Measurement of oxLDL, Lp-PLA2 and lysoPC in plasma of non-smokers and smokers

Plasma circulating oxLDL, collected from whole blood into Vacutainer tubes containing ethylenediamine tetraacetic acid (1 mg/ml) was measured with the enzyme-linked immunosorbent assay Mercodia oxidized-LDL enzyme-linked sandwich immunosorbent assay kit (Mercodia AB, Uppsala, Sweden), in which the wells of the microtiter plates are coated with the capture antibody mAb-4E6 [29]. Cu^2+^-modified LDL ranging from 50 to 500 ng/ml was used as a standard solution. Lp-PLA2 mass was measured using the PLAC test (diaDexus, Inc, San Francisco, California), a microplate-based enzyme immunoassay, based on the principle of an enzyme-linked sandwich immunosorbent assay using 2 highly specific monoclonal antibodies.

LysoPC 16:0 (1-palmitoyl-2-hydroxy-sn-glycero-3-phosphocholine) in plasma was measured on an Agilent mass spectrometer equipped with electrospray ionization ion source. Experiments were performed with a high-performance liquid chromatography-mass spectrometry system (HP1100 ion-trap, Agilent technologies, Waldbronn, Germany); column was a C8 4,6x250 mm (Waters Spherisorb); quantification of the chromatograms peak areas was performed by single ion monitoring scan resolution.

### LDL isolation and oxidation

LDL was isolated from whole blood of healthy volunteers after 12 h of fasting by sequential flotation in NaBr solution containing 1 mg/ml ethylenediamine tetraacetic acid (density 1.019–1.063 g/ml). Immediately before the oxidation incubations, LDL was separated from ethylenediamine tetraacetic acid and from diffusible low molecular mass compounds by gel filtration on PD-10 Sephadex G-25M gel in 10 mmol/l phosphate-buffered saline. Cu^2+^-modified LDL (1.7 mg of LDL protein/ml) was prepared by exposure of LDL to 5 µmol/l CuSO_4_ for 18 h at 37° C, as described previously [30]. The extent of LDL oxidation procedure was determined by evaluating the level of thiobarbituric acid-reactive substances, as reported [30].

### Cell culture

PBMC (3x10^5^/ml, 200 µl/well) from healthy donors were cultured in RPMI 1640 with L-glutamine (GIBCO), as described [5].

Human aortic SMCs were purchased from Gibco Cascade Biologics and cultured in medium 231 according to the manufacturing instructions: in brief, human aortic SMCs were plated in 12-well tissue culture plates at a density of 2.0x10^4^ cells/well and were maintained in SMCs growth medium containing 5% foetal bovine serum, 50 mg/ml gentamicin, and 50 ng/ml amphotericin B at 37° C, 5% CO_2_/95% air, until confluence. Because it has been already demonstrated that proteoglycans synthesis increases significantly in proliferating cells in comparison with quiescent cells [31], cultures were then switched to DMEM supplemented with 20% foetal bovine serum and antibiotics to obtain proliferating cells, or DMEM containing 1% foetal bovine serum and antibiotics to obtain quiescent cells, for 3 days. 

### Preparation of oxidized products of the phospholipid 1-palmitoyl-2-arachidonyl-sn-glycero-3-phosphorylcholine (oxPAPC)

PAPC was oxidized by air exposure for 48 hours and the composition of oxPAPC was analyzed as previously described [29]. 75 mg of oxPAPC yelded about 0.6–0.7 mg/mL (1.01–1.17 mmol/L) of 1-palmitoyl-2-(5-oxovaleroyl)-sn-glycero-3-phosphorylcholine (POVPC) and about 0.38–0.45 mg/mL (0.62–0.74 mmol/L) of 1-palmitoyl-2-glutaroyl-sn-glycero-3-phosphorylcholine (PGPC). The lipids were extracted with chloroform and re-suspended in methanol before addition to the PBMC. 

### Effect of increasing concentrations of native LDL, oxLDL, oxPAPC and of lysoPC on mRNA Lp-PLA2 expression in PBMC

PBMC were incubated with increasing concentrations of native LDL (nLDL) (as control), oxLDL, oxPAPC (0-100 µg/ml) and of lysoPC (0-50 µmol/l) in RPMI containing 0.2% foetal bovine serum for 6 hours at 37° C. Standard lysoPC (16:0) was purchased from Avanti Polar Lipids. 

### Quantification of POVPC, PGPC and lysoPC in PBMC after incubation with nLDL or oxLDL

Oxidized products in PBMC were measured on an Agilent mass spectrometer equipped with an electrospray source as previously described [32,33]. Among the different oxPAPC, POVPC and PGPC were taken into consideration because they have been previously identified as the most bioactive components [6]. For the experiments, PBMC were incubated with nLDL or oxLDL (100 µg/ml) for 18 hours at 37° C.

Flow injection experiments were performed by a high-performance liquid chromatography system as previously described [29]. Quantification of the peak areas was performed by single ion monitoring in the elution time range of 10–20 min using appropriate software. Authentic PAPC, POVPC (593.4 *m/z*) and PGPC (609.4 *m/z*) were obtained from Avanti Polar Lipids, Inc. (Alabaster, AL) [32].

### Effect of increasing concentrations of lysoPC on versican and biglycan (mRNA and protein) expression in human aortic SMCs

Before the experiments, the medium was changed and both quiescent and proliferating human aortic SMCs were incubated without and with increasing concentrations (10-50 µM) of lysoPC in DMEM containing 0.2% foetal bovine serum for 24 hours at 37° C. 

### RNA isolation and quantitative real-time PCR

Total RNA was isolated with QIAzol Lysis reagent and RNEasy Mini Kit (Qiagen, Hilden, Germany). The concentration and quality of RNA were evaluated using the RNA 6000 Nano LabChip Kit (Agilent 2100 Bioanalyzer, Agilent Technologies Inc., Santa Clara, CA, USA). Reverse transcription of total RNA was carried out using IScript cDNA Synthesis Kit (Bio-Rad, Hercules, CA, USA) according to the manufacturer's recommendations. The relative expression levels of genes of interest (Lp-PLA2, biglycan and versican) were performed in triplicate using QuantiTect Primer Assay and QuantiTect SYBR Green PCR Kit (Qiagen, Hilden, Germany) on the MyiQ Thermal Cycler (Bio-Rad, Hercules, CA, USA) as previously described [28]. QuantiTect Hs-ACTB Assay (Qiagen, Hilden, Germany) was used as normalizer.

### Western Blotting

Western blotting analysis was performed as described elsewhere [5,28]. The following primary antibodies against the indicated proteins were used: Lp-PLA2 (ab 96619), versican (ab19345), biglycan (ab 49701) (Abcam, Cambridge, UK), and beta-actin (SC-130656) (Santa Cruz Biotechnology Inc., Santa Cruz, CA, USA). As secondary antibodies, appropriate horseradish peroxidase-conjugated antibodies were used and the bands visualized according to a standard chemiluminescence protocol (Immobilon Western, Millipore, Billerica, MA, USA). Protein expression was quantified using VersaDoc Imaging System (Bio-Rad, Hercules, CA, USA).

### Statistical analysis

Data are expressed as mean±SD values if normally distributed. Differences between two groups were analysed by a two-tailed unpaired Student’s t-test. The relationship between variables was assessed by linear regression. A probability value (P) of 0.05 was considered to be statistically significant. All data were analysed with StatView (SaS).

## Results

### In vivo study

**Figure 1:**
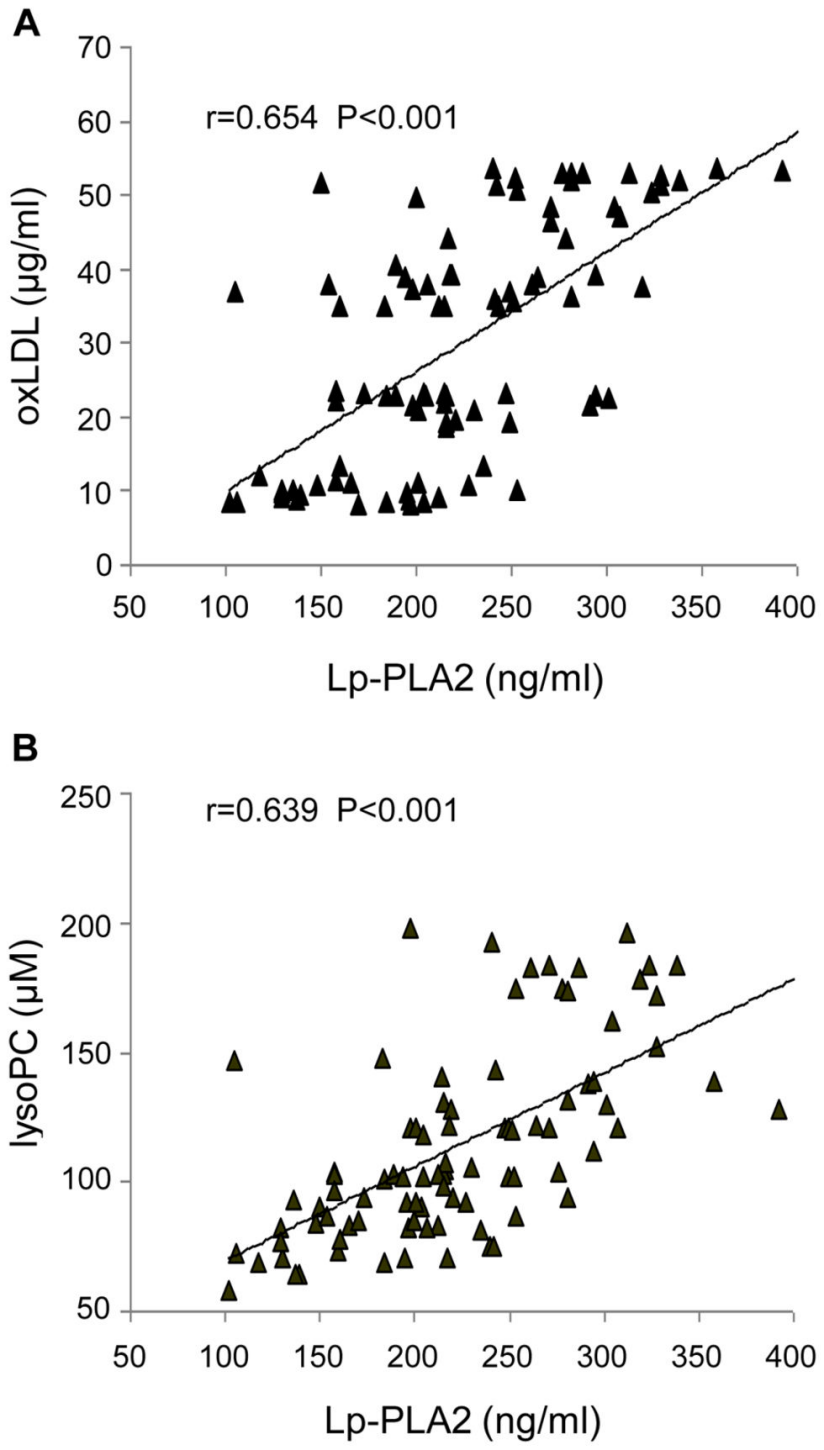
Correlation between plasma lipoprotein-associated phospholipase A2 (Lp-PLA2) and oxidized LDL (oxLDL) and between plasma LpPLA2 and lysophosphatidylcholine (lysoPC). (A) Correlation between plasma Lp-PLA2 and oxLDL and (B) correlation between plasma Lp-PLA2 and lysoPC concentrations in the whole population of subjects studied.

Both groups of non-smokers and smokers were comparable with respect to age and gender; moreover waist circumference, body mass index, systolic and diastolic blood pressure, plasma glucose and lipid profile were within normal range and similar in both groups. In contrast, smokers compared to non-smokers, had significantly higher levels of circulating oxLDL (44.7±9.1 versus 15.8±7.5 µg/ml respectively, P<0.001). Moreover our results showed higher plasma levels of Lp-PLA2 (256.7±63.1 versus 191.3±48.0 ng/ml respectively, P<0.001) and of lysoPC (133.8±39.4 µmol/l and 95.3±20.8 µmol/l respectively, P<0.001) in smokers compared to non-smokers. These data are presented in Table 1. We also found a significant positive correlation between plasma concentrations of ox-LDL and of LpPLA2 (r=0.654, P<0.001) and between plasma concentrations of Lp-PLA2 and of lysoPC in the whole population of subjects studied (r=0.639, P<0.001), (Figure 1, A-B).

**Table 1:** Clinical, metabolic and circulating inflammatory and oxidative stress parameters of non-smokers and smokers.

	**Non-smokers**	**Smokers**	**P**
	**(n = 45)**	**(n = 45)**	
Age (years)	35.1±6.1	36.5±6.4	ns
Male/female	23/22	25/20	ns
BMI (Kg/m^2^)	23.1±2.2	22.4±2.8	ns
SBP (mmHg)	116.4±14.1	118.1±15.5	ns
DBP (mmHg)	72.1±12.7	71.3±11.2	ns
Heart rate (bpm)	65.6±8.3	73.2±7.8	ns
Waist circumference (cm)	79.0±11.9	76.5±10.8	ns
Total cholesterol (mg/dl)	189.7±24.6	186.4±21.3	ns
LDL cholesterol (mg/dl)	115.5±20.1	116.3±19.4	ns
HDL cholesterol (mg/dl)	52.5±11.4	51.4±10.9	ns
Triglycerides (mg/dl)	89.7±27.6	92.9±25.6	ns
Plasma glucose (mg/dl)	79.9±10.4	83.4±9.3	ns
oxLDL (µg/ml)	15.8±7.5	44.7±9.1	0.001
Lp-PLA2 (ng/ml)	191.3±48.0	256.7±63.1	0.001
lysoPC (µM)	95.3±20.8	133.8±39.4	0.001

Legend: BMI= body mass index; SBP= systolic blood pressure; DBP= diastolic blood pressure; oxLDL= oxidized LDL; Lp-PLA2= lipoprotein associated-phospholipase A2; lysoPC= lysophosphatidilcholine.

**Figure 2:**
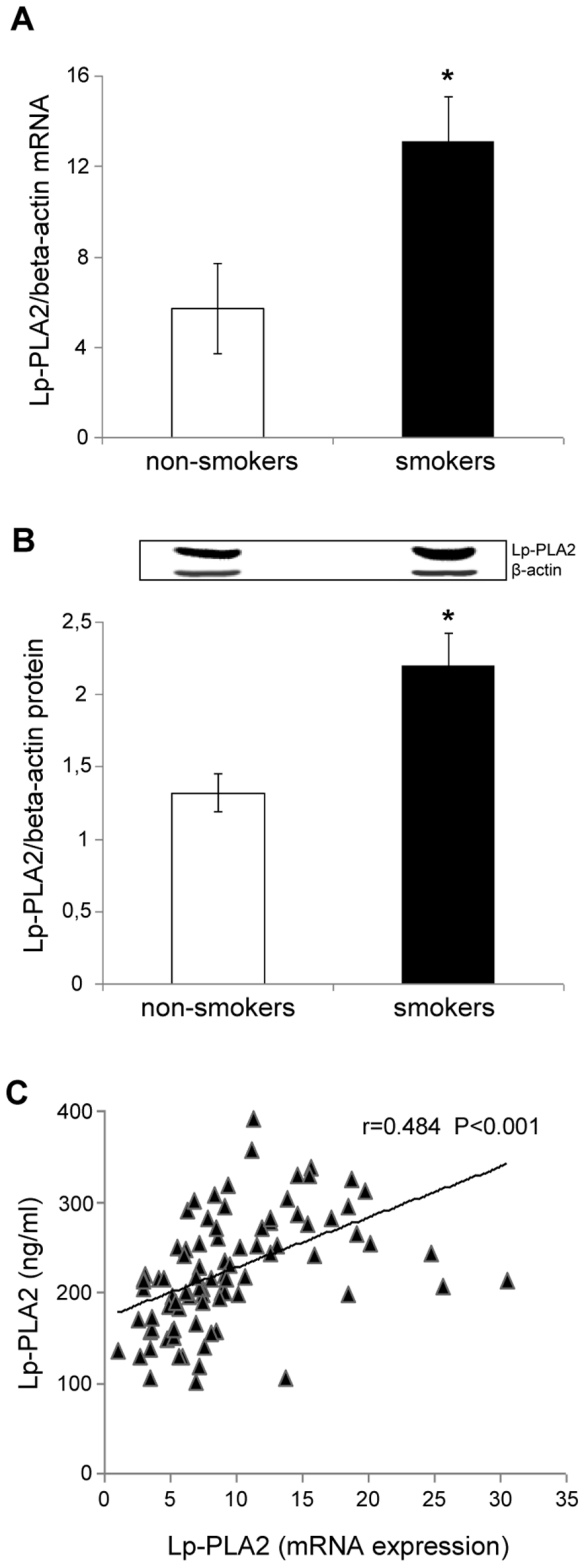
Lipoprotein-associated phospholipase A2 (Lp-PLA2) mRNA and protein expression in peripheral blood mononuclear cells (PBMC) of non-smokers and smokers and correlation between Lp-PLA2 expression in PBMC and plasma Lp-PLA2. (A) mRNA was analyzed by quantitative Real-Time PCR. Normalized gene expression levels were given as the ratio between the mean value for the Lp-PLA2 gene and that for beta-actin in each sample. Data are presented as mean+SD. *P<0.001 versus non-smokers. (B) Representative Western blot analysis for Lp-PLA2 and the average quantification obtained by densitometric analysis of all the samples (20 subjects per group, randomly selected). (C) Correlation between Lp-PLA2 mRNA expression in PBMC and plasma concentrations of Lp-PLA2 in the whole population of subjects studied.

We then examined the expression of Lp-PLA2 in PBMC. As shown in Figure 2, A-B, our results demonstrated a significantly increased expression of Lp-PLA2 (mRNA and protein) in smokers compared to non-smokers. These findings together with the fact that in all subjects we found a positive correlation between the expression of mRNA Lp-PLA2 in PBMC and the plasma concentrations of Lp-PLA2 (r=0.484, P<0.001), (Figure 2C) indicate that in our subjects the expression of Lp-PLA2 in PBMC may be a major source of circulating Lp-PLA2.

**Figure 3:**
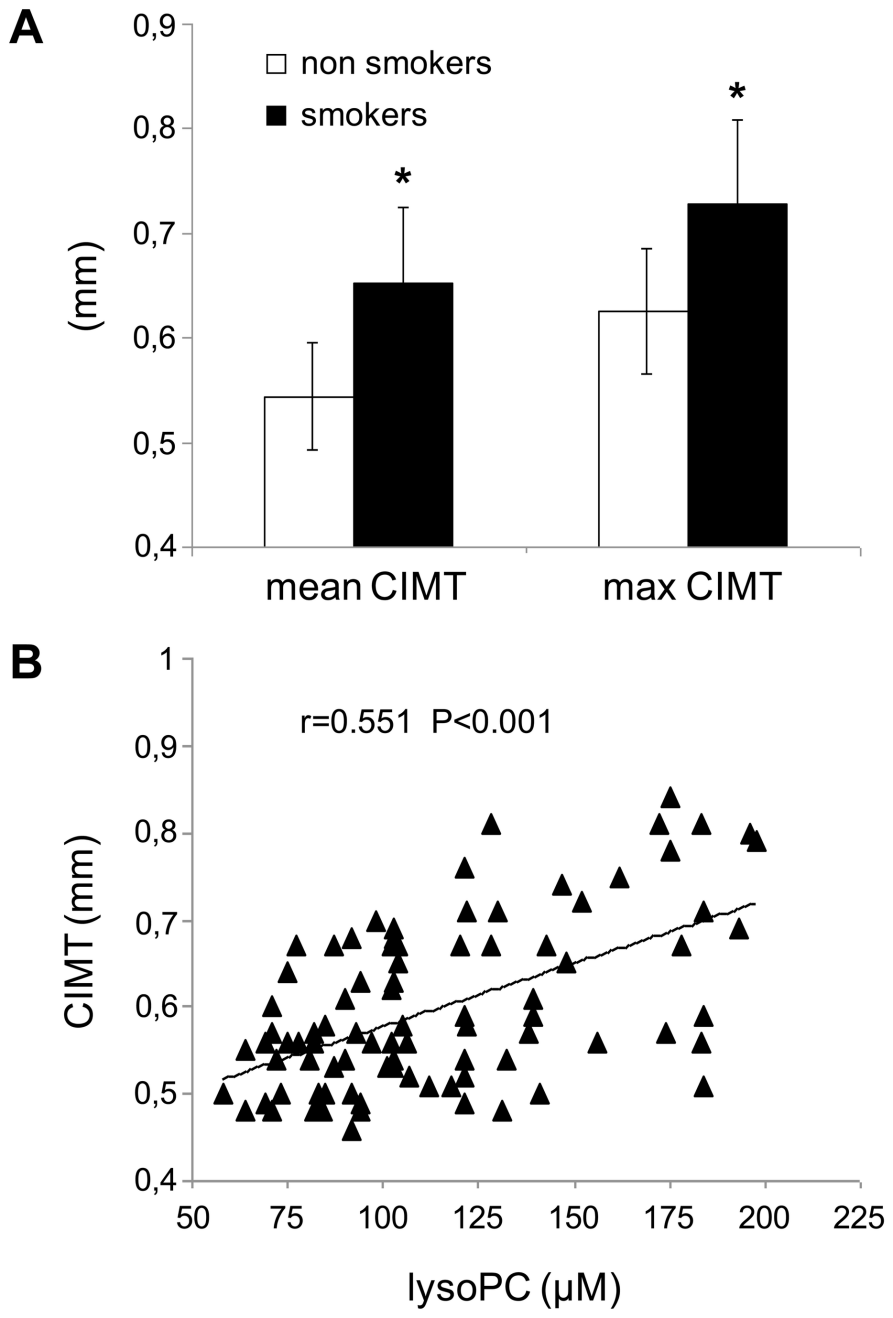
Carotid intima-media thickness (CIMT) in non-smokers and smokers and correlation between CIMT and plasma concentrations of lysophosphatidylcholine (lysoPC). (A) Mean and maximal CIMT values in non-smokers and smokers, *P<0.001 versus non-smokers. Data are presented as mean+SD. (B) Correlation between CIMT and plasma concentrations of lysoPC in the whole population of subjects studied.

To evaluate cigarette smoking-induced early alterations of the arterial wall, CIMT was measured in all the subjects. In smokers mean and maximal CIMT, though in a normal range, were significantly higher than in non-smokers; in particular, mean CIMT was 0.65±0.09 mm in smokers and 0.54±0.06 mm in non-smokers (P<0.001) and maximal CIMT was 0.73±0.10 mm in smokers and 0.63±0.06 in non-smokers (P<0.001), as shown in Figure 3A. Interestingly, our results also showed a positive correlation between mean CIMT and lysoPC (r=0.551, P<0.001) in all the subjects (Figure 3B). Furthermore a significant positive correlation was also demonstrated between plasma oxLDL and mean CIMT (r=0.543, P<0.001) and between Lp-PLA2 and mean CIMT (r=0.382, p<0.001) in all the subjects. Finally, there was no evidence of carotid plaques in any subject; this finding was not unexpected given their relatively young age and the absence of other cardiovascular risk factors.

### In vitro study

#### OxLDL- and oxPAPC-induced Lp-PLA2 expression in PBMC

**Figure 4:**
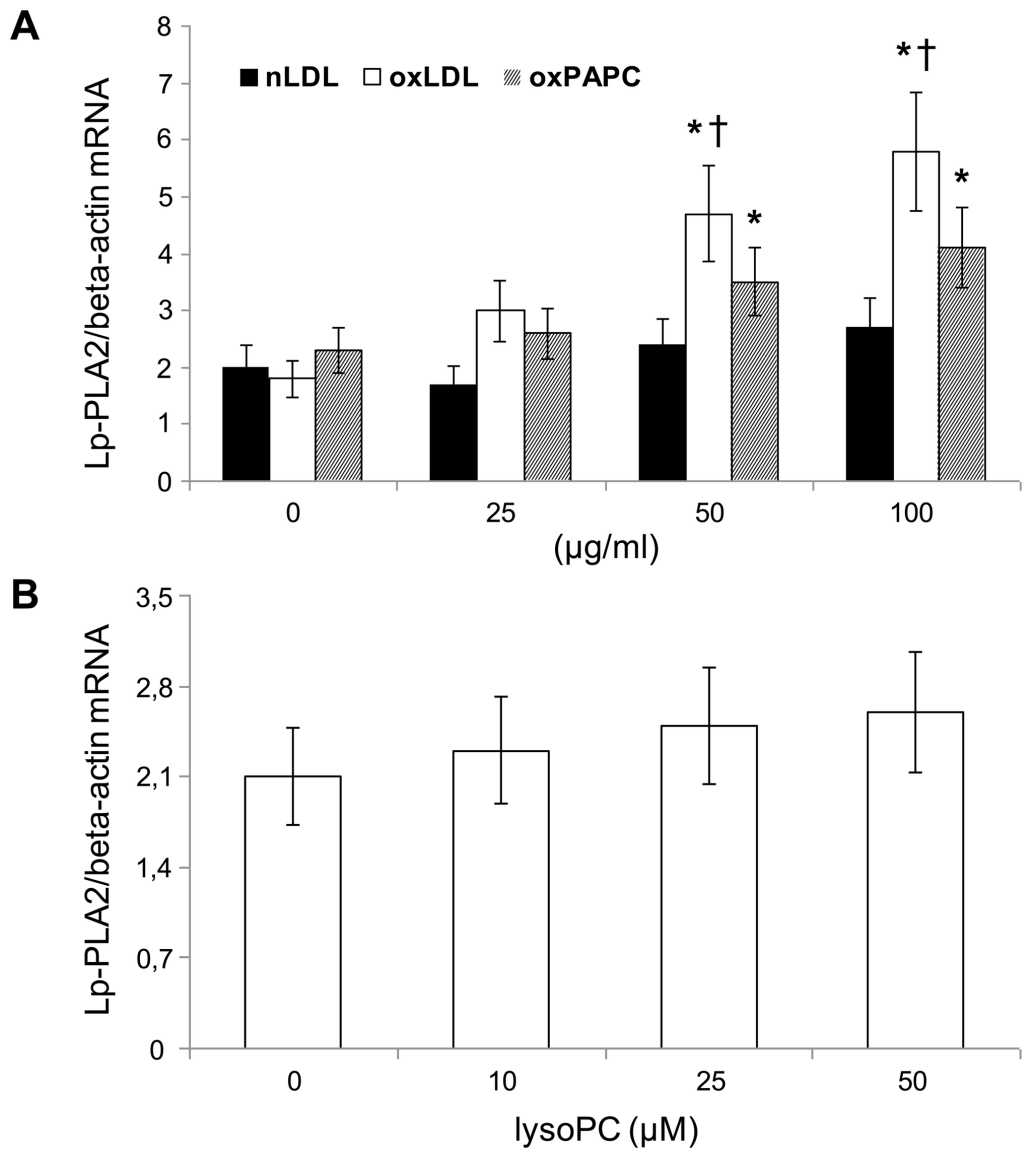
Dose-response effect of native LDL (nLDL), oxidized LDL (oxLDL), oxidized phospholipids (oxPAPC), and of lysophosphatidylcholine (lysoPC) on mRNA expression of Lp-PLA2. (A) Dose-response effect of nLDL, oxLDL and oxPAPC (0-100 µg/ml) and (B) dose-response effect of lysoPC (0-50 µM) on mRNA expression of Lp-PLA2 after incubation with PBMC for 6 h. Normalized gene expression levels were given as the ratio between the mean value for Lp-PLA2 gene and that for the beta-actin in each sample. Data represent the mean±SD of measurements performed in triplicate in four different occasions. *P<0.01 versus basal, †P<0.01 versus oxPAPC.

Next we tested the hypothesis that circulating oxLDL, and in particular the oxPAPC contained in oxLDL, may have a role in up-regulating the expression of Lp-PLA2 in PBMC. Our results showed that oxLDL (at concentrations similar to those found in smoker's serum) and oxPAPC induced a dose-dependent increase (P<0.01 starting from 50 µg/ml) of Lp-PLA2 (mRNA expression), whereas nLDL and lysoPC did not (Figure 4 A-B). Moreover the mRNA expression of Lp-PLA2 induced by oxLDL was significantly higher than that induced by oxPAPC (P<0.01).

**Figure 5:**
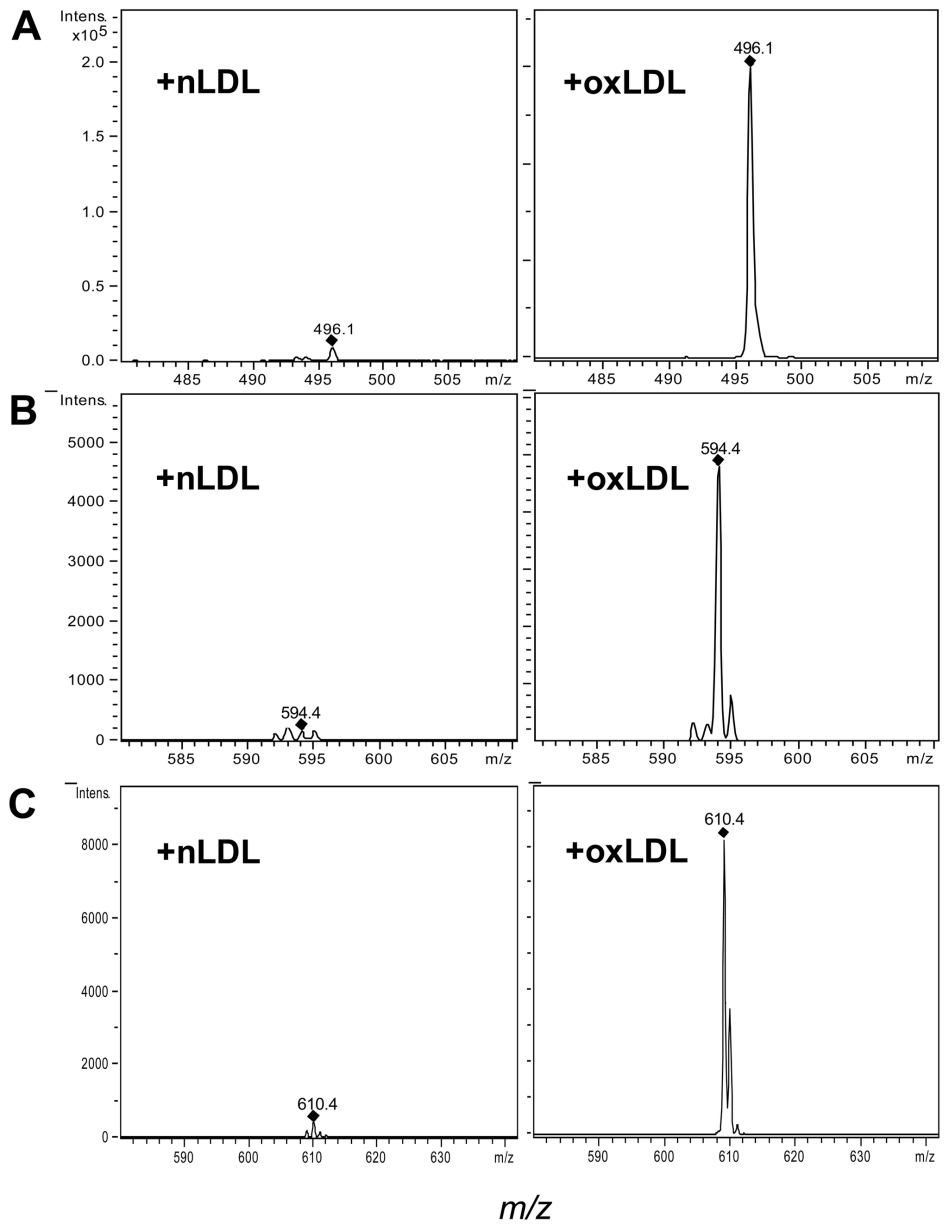
Representative mass spectrometry (MS) analysis of PBMC extract. PBMC were incubated with native LDL (nLDL) or oxidized LDL (oxLDL) (100 µg/ml) for 18 h. Indicated peaks correspond to A) lysoPC (496.1 *m/z*), B) POVPC (594.4 *m/z*) and C) PGPC (610.4 *m/z*). MS analysis of PBMC extract (1/10 with 10 mM ammonium acetate in methanol) was performed on an ion trap mass spectrometer where ions were generated with an electrospray ionization source at 350° C, and spectra were acquired in the positive mode (total ion range 480–640 *m/z*).

Therefore, to clarify whether this up-regulation of mRNA Lp-PLA2 could be due, at least in part, to the ability of oxLDL to transfer and/or generate oxidized phospholipids on PBMC membranes, we quantified the content of POVPC, PGPC and of lysoPC after incubation of PBMC with nLDL or oxLDL. Our results showed that oxLDL-treated PBMC contained more oxPAPC (POVPC and PGPC) and lysoPC that nLDL-treated PBMC. Figure 5 A-C shows a representative mass spectometry analysis of POVPC, PGPC and of lysoPC content after incubation of PBMC in presence of nLDL and oxLDL. 

#### Effect of increasing concentrations of lysoPC on biglycan and versican (mRNA and protein) expression in human aortic SMCs

**Figure 6:**
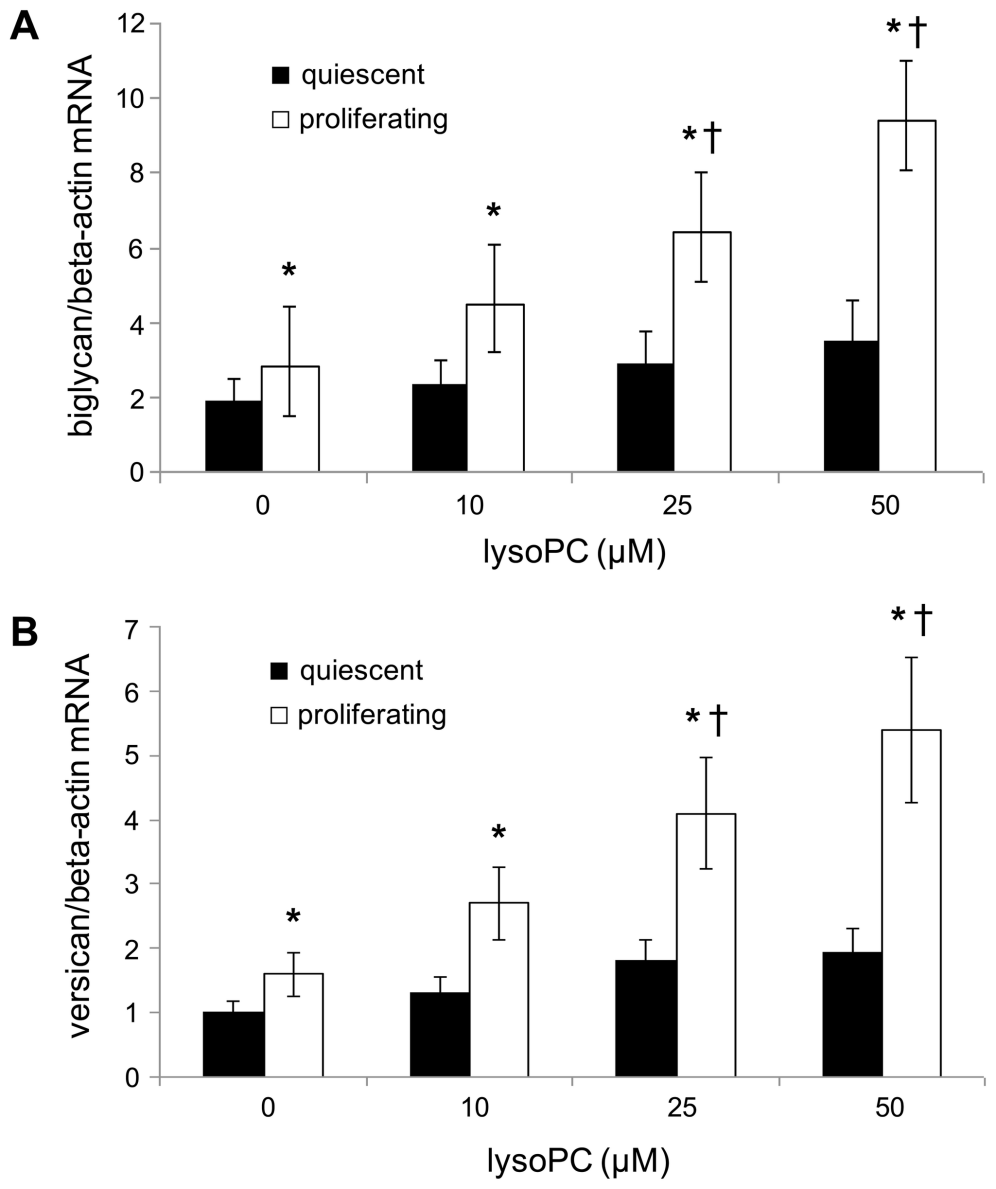
Dose-response effect of lysophosphatidylcholine (lysoPC) on biglycan and versican mRNA expression. Effect of increasing concentrations (0-50 µM) of lysoPC on (A) biglycan and on (B) versican mRNA expression after incubation with quiescent and proliferating human aortic SMCs for 24 h. Normalized gene expression levels were given as the ratio between the mean value for target gene and that for the beta-actin in each sample. Data represent the mean±SD of measurements performed in triplicate in four different occasions. * P<0.01 versus quiescent; †P<0.01 versus basal.

**Figure 7:**
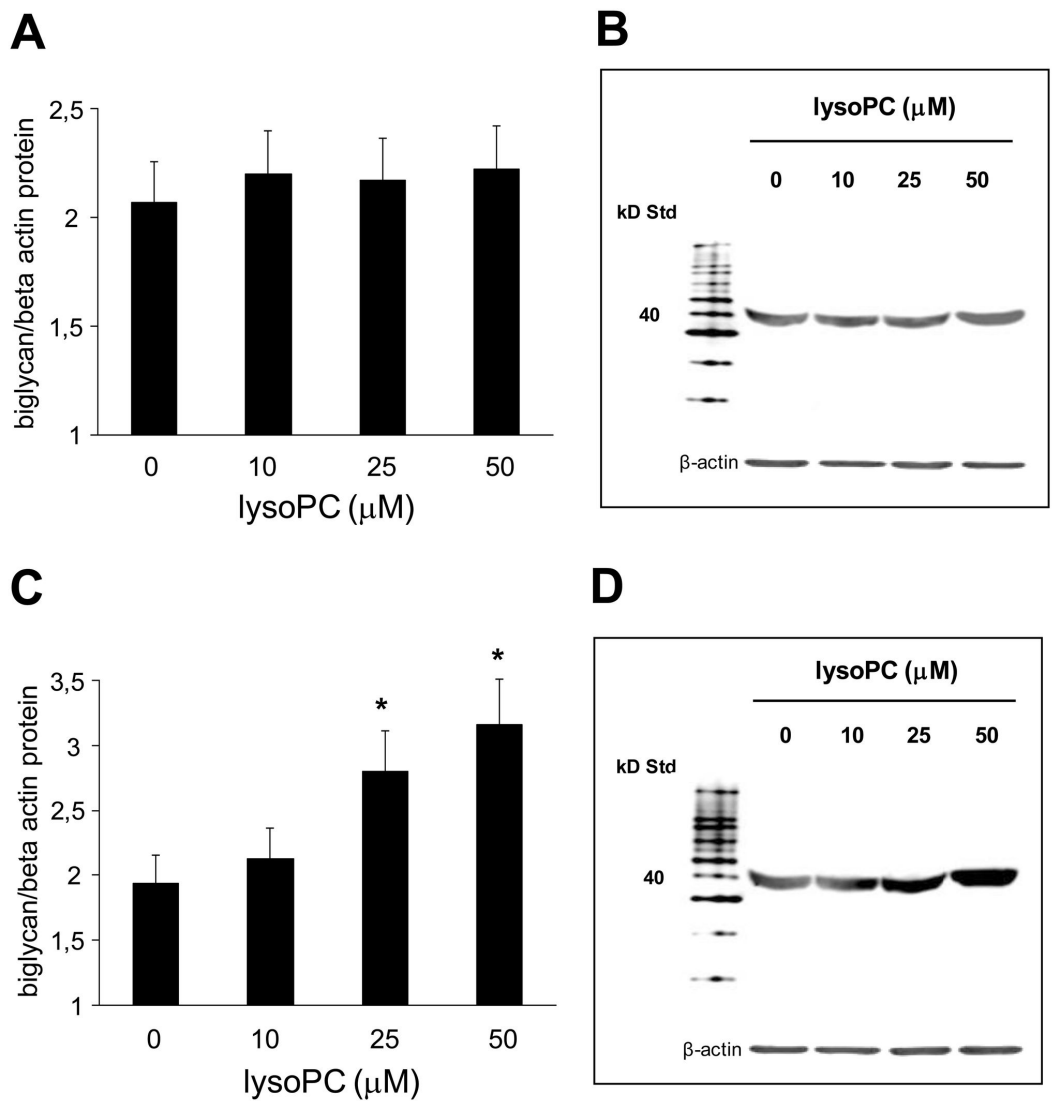
Dose-response effect of lysophosphatidylcholine (lysoPC) on biglycan protein expression. Effect of increasing concentrations (0-50 µM) of lysoPC on biglycan protein expression after incubation with (A) quiescent and (C) proliferating human aortic SMCs for 24 h. Data are the average quantification obtained by densitometric analysis of all the samples, expressed as the density ratio of target to control (beta-actin) in arbitrary units x10. Figure also shows a representative Western blot analysis (B and D) of three independent experiments for biglycan.

Moreover, we exposed quiescent and proliferating human aortic SMCs to increasing concentrations of lysoPC and evaluated the expression of biglycan and versican. As shown in Figure 6 A-B, our results indicated that the expression of both biglycan and versican was significantly higher in proliferating compared to quiescent human aortic SMCs; however and more interestingly, both biglycan and versican mRNA expression was dose-dependently increased in human aortic SMCs incubated with lysoPC (P<0.01 starting from 25 µmol/l). In parallel with mRNA, similar results were also found for the protein expression of biglycan (Figure 7A-D) and versican (data not shown). 

## Discussion

Oxidative stress induced by cigarette smoking plays a central role in accelerated cardiovascular aging in smokers [4]. In this study, we first showed higher levels of oxLDL in smokers compared to non-smokers, confirming previous studies that demonstrated an increase of oxidative stress markers in smokers [4,5,34]. Even if augmented levels of Lp-PLA2 have been observed in smokers [35,36], this is the first demonstration of higher plasma levels of Lp-PLA2 and of lysoPC in young healthy smokers compared with age- and sex-matched non-smokers. Although it could be argued that in this study we measured only Lp-PLA2 mass levels, there are convincing epidemiological data showing that Lp-PLA2 concentrations can be assessed by either enzyme activity or enzyme mass measurements [7]. Moreover, the fact that in both groups the HDL and LDL cholesterol levels were normal and comparable, allows us to reasonably exclude that Lp-PLA2 levels may depend on different lipoprotein carrier concentration in plasma. On the contrary, the positive correlation between Lp-PLA2 and oxLDL found in all the subjects, suggests that oxLDL may influence plasma levels of Lp-PLA2. Furthermore our results also showed a positive correlation between plasma Lp-PLA2 and lysoPC, indicating that Lp-PLA2 may be a major determinant of lysoPC plasma levels. 

Several studies show that the activation of leukocytes plays a crucial role in atherogenesis and that Lp-PLA2 is mainly secreted by inflammatory cells in circulation or in the atherosclerotic plaques [7]. In this study we also demonstrated a positive correlation between mRNA expression of Lp-PLA2 in PBMC and plasma Lp-PLA2 levels in all the subjects; this finding is in line with a recent study showing that in non-obese women plasma Lp-PLA2 activity positively correlated with PBMC Lp-PLA2 activity [37]. Moreover, given the strong association between carotid and coronary atherosclerosis [12,13], the evidence that in no subject carotid plaques were detected, supports the conclusion that PBMC are a major source of Lp-PLA2 in our young healthy subjects. 

Furthermore, another peculiarity of this study is the increased expression (mRNA and protein) of Lp-PLA2 in PBMC derived from smokers. Even though epidemiological studies have demonstrated an association between cigarette smoking and Lp-PLA2 activity [35,36], whether the oxidative stress associated with cigarette smoking may have a causal role is still not known. Since oxLDL contain different oxidized phospholipids, some of which are thought to act as intracellular signaling molecules [38], in this study we tested the hypothesis that circulating oxLDL may up-regulate the mRNA expression of Lp-PLA2 in PBMC. In particular the role of oxPAPC as intracellular signaling molecules beside the function of membrane phospholipid metabolites is becoming increasingly clear [38]. In this context we have already demonstrated an increased formation of oxPAPC in PBMC of smokers [5], together with their potential to increase the expression of inflammatory genes [5] and to decrease the expression of antioxidant genes [5,6]. In this study we go further and demonstrate that oxLDL and oxPAPC, but not nLDL, dose-dependently increased mRNA expression of Lp-PLA2 in PBMC from healthy donors. A likely explanation of this finding may be that these major oxidized components of oxLDL are transferred or induced on PBMC membranes. The evidence obtained by mass spectrometry that the content of oxPAPC in PBMC was significantly increased after exposure of oxLDL supports the hypothesis that oxPAPC may have a causal role in up-regulation of Lp-PLA2 mRNA expression in PBMC.

Moreover the fact that the concentrations of oxLDL stimulating PBMC Lp-PLA2 expression *in vitro* were similar to those found in smoker’s plasma supports the hypothesis that plasma oxLDL may play an important role in up-regulating Lp-PLA2 mRNA expression in PBMC of smokers. This finding, albeit in different experimental conditions, is in line with a recent paper showing that a synthesized oxidized phospholipid was able to up-regulate Lp-PLA2 mRNA expression in THP-1 cells [10], and also with a porcine model of diabetes demonstrating up-regulation of Lp-PLA2 mRNA expression by PBMC in the presence of advanced glycation end products [39]. As far as the possible mechanism by which oxLDL induces Lp-PLA2 expression is concerned, it has been shown that oxLDL and more specifically its oxidized phospholipids, can up-regulate Lp-PLA2 expression in THP-1 cells through activation of phosphatidylinositol 3-kinase and p38 mitogen-activating protein kinase pathway [10]. 

A further result of this study is the demonstration of higher CIMT values in healthy smokers than in age-sex matched non-smokers.This finding is in accordance with epidemiological studies that have consistently demonstrated, albeit in older subjects, that cigarette smoking is associated with higher CIMT [16,17]. Although convincing evidence has demonstrated a relationship between Lp-PLA2 and incident cardiovascular disease, the association between Lp-PLA2 and CIMT is less clear [7] and probably not independent of other cardiovascular risk factors [40,41]. LysoPC is considered one of the major determinants of the atherosclerotic properties of oxLDL [8,11], and it has been shown to induce several key steps in the development of atherosclerosis [8,11]. Interestingly in this study we demonstrated for the first time a positive correlation between CIMT and plasma lysoPC in all the subjects. In this context, recent data suggest that cigarette smoking has a specific fibrogenic effect which causes intimal thickening, a lesion composed of smooth muscle cells and proteoglycans/collagen matrix with rare inflammatory cells [20-22]. The proteoglycans and specifically biglycan and versican, that are predominant vascular extracellular matrix proteoglycans, are known to play a pivotal role in early atherogenesis [19]. In this regard, recent observations have shown that the initial entrapment of LDL within the extracellular matrix depends on interaction of LDL with biglycan and versican [20-22] and atherosclerosis-prone arteries, such as the coronary artery, have a thickened intima enriched in versican and biglycan [20-22]. Therefore to better understand the possible causal role of lysoPC in the higher CIMT found in smokers, we performed an *in vitro* study aimed to assess whether lysoPC may influence proteoglycans synthesis. Our results support this hypothesis. In fact we demonstrated both an increased expression of biglycan and versican in proliferating compared to quiescent human aortic SMCs and a lysoPC dose-dependent increase in the expression of biglycan and versican in human aortic SMCs. Our results, though in different experimental conditions, are in agreement with previous data showing that lysoPC was able to increase proteoglycans synthesis in SMCs [24].

In conclusion, in our otherwise healthy smokers a further effect of raised oxidative stress is up-regulation of Lp-PLA2 expression in PBMC with subsequent increase of plasma Lp-PLA2 and lysoPC. Moreover the correlation between lysoPC and CIMT together with the finding that lysoPC up-regulates proteoglycan synthesis suggests that lysoPC may be a link between smoking and intimal thickening.
